# Development and Study of Nanoemulsions and Nanoemulsion-Based Hydrogels for the Encapsulation of Lipophilic Compounds

**DOI:** 10.3390/nano10122464

**Published:** 2020-12-09

**Authors:** Sotiria Demisli, Evgenia Mitsou, Vasiliki Pletsa, Aristotelis Xenakis, Vassiliki Papadimitriou

**Affiliations:** 1 Institute of Chemical Biology, National Hellenic Research Foundation, 11635 Athens, Greece; sdemisli@eie.gr (S.D.); emitsou@eie.gr (E.M.); vpletsa@eie.gr (V.P.); arisx@eie.gr (A.X.); 2 Department of Biochemistry & Biotechnology, University of Thessaly, 41500 Larissa, Greece

**Keywords:** nanoemulsions, hydrogels, vitamin D_3_, curcumin, in vitro release, cell viability, DLS, EPR

## Abstract

Biocompatible nanoemulsions and nanoemulsion-based hydrogels were formulated for the encapsulation and delivery of vitamin D_3_ and curcumin. The aforementioned systems were structurally studied applying dynamic light scattering (DLS), electron paramagnetic resonance (EPR) spectroscopy and viscometry. In vitro studies were conducted using Franz diffusion cells to investigate the release of the bioactive compounds from the nanocarriers. The cytotoxicity of the nanoemulsions was investigated using the thiazolyl blue tetrazolium bromide (MTT) cell proliferation assay and RPMI 2650 nasal epithelial cells as in vitro model. DLS measurements showed that vitamin D_3_ and curcumin addition in the dispersed phase of the nanoemulsions caused an increase in the size of the oil droplets from 78.6 ± 0.2 nm to 83.6 ± 0.3 nm and from 78.6 ± 0.2 nm to 165.6 ± 1.0 nm, respectively. Loaded nanoemulsions, in both cases, were stable for 60 days of storage at 25 °C. EPR spectroscopy revealed participation of vitamin D_3_ and curcumin in the surfactants monolayer. In vitro release rates of both lipophilic compounds from the nanoemulsions were comparable to the corresponding ones from the nanoemulsion-based hydrogels. The developed o/w nanoemulsions did not exhibit cytotoxic effect up to the concentration threshold of 1 mg/mL in the cell culture medium.

## 1. Introduction

In recent years, many research groups worldwide have focused their attention on the development of biocompatible systems at the nanoscale for the effective encapsulation, protection and delivery of sensitive bioactive ingredients [1,2,3,4].

Among different nanosized systems, nanoemulsions based on biological amphiphiles and different oils of natural origin, have been studied by several research groups as encapsulation media of active compounds with potential application in foods, pharmaceutics and cosmetics [5,6,7,8,9]. Nanoemulsions are colloidal dispersions of two immiscible liquids stabilized by surfactant molecules. They are kinetically stable and can be obtained even at low concentrations of surfactants, thus being important for various industry sectors. Nanoemulsions are transparent, translucent or milky depending on their droplet sizes. Nanoemulsions are classified into two types, water-in-oil (w/o) and oil-in-water (o/w), according to the proportion and chemical nature of the components. For their preparation, either high or low-energy emulsification methods are applied. The very small droplet size of nanoemulsions (generally in the size range 20–500 nm) makes them resistant to physical destabilization, flocculation and creaming [10,11].

Nanoemulsions formulated with biocompatible and nontoxic ingredients are proposed for numerous applications related to the administration of drugs or other bioactive compounds through various administration routes. In particular, o/w nanoemulsions are effective systems for encapsulating and delivering lipophilic active components. In fact, encapsulation at the nanoscale enhances the solubility, stability and biological activity of the bioactive compounds. However, due to their low viscosity, nanoemulsions cannot be easily applied for topical administration. Besides biomedical applications, incorporation of various lipophilic bioactive agents into functional food gels is not possible due to the fluidity of the loaded nanoemulsions. To address these issues, it has been proposed to integrate o/w nanoemulsions loaded with lipophilic bioactive compounds into hydrogels [12]. Hydrogels are three-dimensional networks of cross-linked hydrophilic polymers. Due to the presence of hydrophilic functional groups attached to the polymeric backbone, they can absorb large amounts of water as well as biological fluids. Hydrophilic gels can be formulated using either synthetic or naturally occurring materials including gelatine, chitosan, xanthan gum, and other cellulose derivative polymers [12,13,14]. Nanoemulsion-based hydrogels have been suggested as delivery systems for various lipophilic bioactive compounds including rosmarinic acid [15], cumin essential oil [16], pentyl gallate [17], genistein [18], and thyme oil [19]. Hydrogels containing loaded nanoemulsions could be incorporated into various functional foods or pharmaceutical formulations intended to be used for topical application.

Vitamin D_3_ (cholecalciferol) is the most biologically active and chemically stable form of vitamin D, essential for the proper functioning of the organism and also vital for the prevention of many chronic diseases. In recent years, numerous research studies have highlighted the strong effect of vitamin D_3_ on brain function, acting through specific receptors present in various areas of the brain [20]. Vitamin D_3_, a steroid hormone, is soluble in fats, oils and several organic solvents but insoluble in water. Since vitamin D_3_ is easily degraded losing its functionality and health benefits and also exhibits poor water solubility and oral bioavailability, nanoencapsulation in appropriate biocompatible carriers could be beneficial for any potential application in the food and pharmaceutical sector. The higher efficacy of nano-encapsulated vitamin D_3_ compared to its free form suggests potential utility of various nanocarriers as a new therapeutic strategy, including oral, transdermal and intranasal administration [21,22,23,24]. In particular, transdermal delivery of therapeutic amounts of vitamin D_3_ using nanocarriers has been proposed as most promising regarding the overcome of side effects on the gastrointestinal tract, providing continuous drug delivery, and being easy to apply or remove [25,26]. It should be emphasized that in the emerging field of pharmaceutical nanotechnology, nasal delivery of vitamin D3 and other drugs using biocompatible nanocarriers has so far received less attention compared to other routes of administration [27].

Curcumin is a naturally derived polyphenol with a variety of pharmacologic properties. Curcumin has been shown to possesses anti-inflammatory, antioxidant, chemopreventive and chemotherapeutic activity [28,29]. Although research data clearly demonstrate the medicinal properties of curcumin, its use as an effective therapeutic agent is still limited due to its extremely low solubility in water and oils, poor targeting, low selectivity and rapid degradation [30]. In order to address these problems, nanoencapsulation of curcumin in selected carriers is proposed as a promising and effective alternative technique [31]. Furthermore, intranasal delivery of nanoencapsulated curcumin in biocompatible carriers for brain targeting is an innovative strategy for the effective delivery of this natural polyphenolic compound [32].

The aim of this work was to develop and characterize (a) nanoemulsions and (b) nanoemulsion-based hydrogels as carriers of lipophilic compounds. For this purpose, a series of o/w nanoemulsions based on water as the continuous phase, biocompatible oils as the dispersed phase and safe for human use surface-active molecules were developed. To formulate the o/w nanoemulsions, either low- or high-energy emulsification procedures were applied. Following this, selected nanoemulsions were used for the encapsulation of vitamin D_3_ and curcumin. Loaded nanoemulsions were added in homopolymeric and copolymeric hydrogels based on polysaccharides and their combination [14]. Both o/w nanoemulsions and nanoemulsion-based hydrogels were structurally characterized to evaluate the effect of composition and emulsification process on their properties [33,34]. The ability of the proposed nanoemulsions and nanoemulsion-based hydrogels to release the encapsulated active ingredients in an aqueous environment was also evaluated [35]. Furthermore, the in vitro cytotoxic effect of the nanoemulsions on nasal epithelial cells was investigated.

## 2. Materials and Methods

### 2.1. Materials

Polyoxyethylenesorbitan monooleate (Tween 80), pure, was purchased from Fisher BioReagents, NH, USA. Soybean lecithin (L-alpha phosphatidylcholine), 90% was from Alpha Aesar, Germany. Glycerol monolinoleate (Maisine^®^ CC) HLB 1, Diethylene glycol monoethyl ether (Transcutol^®^ HP) and Caprylocaproyl Polyoxyl-8 glycerides (Labrasol^®^) HLB 12, were kindly donated by Gattefossé, France. Extra virgin olive oil (EVOO) (acidity <0.4%) from the Amfissa Variety, Pelion, Magnesia, Greece, was kindly provided by “Myrolion” start-up company, Greece. Isopropyl tetradecanoate (IPM), 98% was from Αlpha Aesar, Germany. 5- and 16-Doxyl stearic acid, free radicals were from Sigma-Aldrich Chemie Gmbh Munich, Germany. Curcumin was a product of Sigma–Aldrich Co; St. Louis, MO, USA. Chitosan (200–600 mPa·s, 0.5% in 0.5% Acetic Acid at 20 °C) was purchased from TCI, Belgium. Xanthan gum from Xanthomonas campestris and Hydroxypropyl methylcellulose (HPMC) were supplied from Sigma-Aldrich, Chemie Gmbh Munich, Germany. Dulbecco’s Modified Eagle’s Medium (DMEM), foetal bovine serum (FBS), 100 U/mL penicillin and 100 mg/mL streptomycin, phosphate-buffered saline (PBS) and trypsin-EDTA were purchased from Gibco (Life Technologies, Grand Island, NY, USA). Acetic Acid glacial 100% was from Merck, Darmstadt, Germany. Ethanol absolute, analytical grade was from Fisher Scientific, Loughborough, UK. Deionized water was used.

### 2.2. Methods

#### 2.2.1. Preparation of Nanoemulsions

Low-energy method: O/w nanoemulsions (L1, L2) were prepared using EVOO, IPM, Labrasol, Tween 80, Maisine, Transcutol and water. Initially, appropriate amounts of surfactants and oils were mixed under mild stirring at room temperature for 30 min. Then the mixtures were titrated with water under constant magnetic stirring.

High-energy method: O/w nanoemulsions (H1, H2) were prepared in a two-step emulsification procedure using EVOO, IPM, Tween 80, Lecithin, Labrasol, Maisine, Transcutol and water. Coarse emulsions were initially obtained by mechanical agitation at room temperature. Formulation of nanoemulsions was achieved by passing the coarse emulsions through a Panda PLUS1000 (GEA, Niro Soavi, Parma, Italy) high-pressure homogenizer. After 12 recirculation passages at a pressure up to a maximum of 700 bar, the obtained nanoemulsions were immediately cooled using an ice bath.

After preparation, all nanoemulsions (L1, L2, H1, H2) were visually checked and stored in screw-capped glass vials at room temperature. The composition of the nanoemulsions is shown in Table 1.

Nanoemulsions loaded with lipophilic compounds were obtained by dissolving the compound (vitamin D_3_, curcumin) in IPM to result a 3 mg/mL solution. Then, this solution was mixed with the other components to result o/w nanoemulsions as described above.

#### 2.2.2. Preparation of Hydrogels

Hydrogels were formed either using a single type of monomer (homopolymeric) or two different types of monomers (copolymeric). The composition of the hydrogels was the following: (a) Chitosan in acetic acid 1% pH 3, (b) HPMC in water, (c) xanthan gum in water, (d) chitosan/HPMC (1:1) in acetic acid 1% pH 3, (e) chitosan/xanthan gum (1:1) in acetic acid 1% pH 3 and (f) HPMC/xanthan gum (1:1) in water. The method of hydrogel preparation was the following. Appropriate amount(s) of polymer(s) was weighted in a glass tube. Then the dispersion medium was added, and the mixture was gently stirred using a spatula until the polymer was evenly distributed. For the development of the copolymeric hydrogels (combination of two polymers), the polymers were weighted in a ratio 1:1 and were mixed with the appropriate dispersion medium with a spatula.

Nanoemulsion-based hydrogels were formed by adding different amounts of o/w nanoemulsions to hydrogels prepared as mentioned above. Nanoemulsions were incorporated into the hydrogels by gentle stirring with a spatula.

#### 2.2.3. Viscosity Measurements

The Brookfield DV-I Prime Digital Viscometer (Brookfield Engineering Laboratories, Middleboro, MA, USA) was used for the viscosity measurements of the nanoemulsions at given shear rate (7.5 s^−1^). The viscometer was equipped with cone spindle CPA-40Z (Brookfield Engineering Laboratories, Middleboro, MA, USA). The temperature was kept constant at 25 °C using a water bath. Experiments were performed in triplicate and results are presented as mean value ± standard deviation (S.D).

#### 2.2.4. Dynamic Light Scattering (DLS) Measurements

A Zetasizer Nano ZS (ZEN3600) analyser (Malvern Instruments Ltd., Malvern, UK) equipped with a He-Ne laser (633 nm) and Non-Invasive Backscatter (NIBS) optics was used for particle size, PdI and ζ-potential measurements of the o/w nanoemulsions. Results were processed with the Malvern Zetasizer Nano software, version 6.32 (Malvern Instruments Ltd., Malvern, UK) which fits a spherical model of diffusing particles with low polydispersity. Before each measurement, samples were filtered through 0.45 μm hydrophilic PTFE filters (Millex-LCR from Millipore Merck KGaA, Darmstadt, Germany) and contained in dust free quartz cuvettes. Measurements were carried out in triplicate at 25 °C. Results are expressed as mean ± standard deviation (SD).

#### 2.2.5. Stability Study

The physical stability of empty and loaded nanoemulsions was evaluated in terms of droplet size and PdI by DLS measurements using the Zetasizer NanoZS analyzer (ZEN3600) (Malvern Instruments Ltd., Malvern, UK). All o/w nanoemulsions were contained in sealed vials and stored in the dark at constant temperature (25 °C). Measurements were carried out the day of their preparation and at given time intervals. Results are expressed as mean ± SD.

#### 2.2.6. Electron Paramagnetic Resonance (EPR) Spectroscopy Measurements

EPR is a spectroscopic technique that measures the absorption of microwaves by paramagnetic centres with one or more unpaired electrons. EPR spectroscopy plays an important role in the understanding of molecular structure near species with unpaired electrons. In this study, 5- and 16-doxyl stearic acids, two fatty acid derivatives labelled at different positions of the aliphatic chain, were used to probe membrane dynamics at different depths.

EPR measurements were performed with an EMX EPR spectrometer (Bruker BioSpin GmbH, Rheinstetten, Germany) operating at the X-Band (9.8 GHz) using a quartz flat aqueous sample cell (Wilmad-LabGlass, Cortecnet Europe, Voisins-Le-Bretonneux, France). Instrument settings were center field 0.348 T, scan range 0.01 T, receiver gain 5.64 × 10^4^, time constant 10.24 ms, conversion time 5 ms, modulation amplitude 0.4 mT. Sample preparation was as follows: (a) initially, concentrated solutions (0.04 M) of the spin probes were prepared in IPM, (b) nanoemulsions and nanoemulsion-based hydrogels were formulated using the concentrated solutions of the spin probes as the oil phase.

The Bruker WinEPR acquisition and processing program (Bruker BioSpin GmbH, Germany) was used for data collection and analysis. EPR spectra were analysed in terms of rotational correlation time (τ_R_), order parameter (S) and isotropic hyperfine coupling constant (α_N_), as described in detail elsewhere [31,33,34].

#### 2.2.7. In Vitro Release Study

The in vitro release of encapsulated vitamin D_3_ and curcumin from nanoemulsions and nanoemulsion-based hydrogels was investigated using vertical glass Franz diffusion cells (PermeGear Inc. Bethlehem, Hellertown, PA, USA). The effective diffusion surface area of each cell was 0.785 cm^2^ (1 cm diameter orifice). Each cell consisted of a donor compartment and a receptor compartment separated by a synthetic cellulose membrane with molecular weight cut-off 14,000 and average flat width 43 mm (Sigma-Aldrich Co St. Louis, MO, USA). The membrane was previously left for 24 h in a 1:1 PBS buffer/ethanol solution in order to hydrate. The cells were water-jacketed and connected to a thermostatically controlled water bath (Haake DC10, Karlsruhe, Germany). The receptor compartments were filled with 5 mL of the 1:1 PBS buffer/ethanol solution and magnetically stirred throughout the experiment. The presence of ethanol in the receiver solution is crucial for the solubility of vitamin D3 and curcumin in the aqueous phase. The experiments were carried out at 32 ± 0.5 °C. The permeated amounts of vitamin D_3_ and curcumin in the receiver solution at different sampling times were calculated by a Cary UV-VIS spectrophotometer (Agilent, Santa Clara, CA, USA) at the wavelength of peak absorption using the following standard curves: Vitamin D_3_ (259 nm) y = 0.037x, R^2^ = 0.993 Curcumin (213 nm) y = 0.158x, R^2^ = 0.999. To obtain the membrane permeation profiles of vitamin D_3_ and curcumin from the nanocarriers, the cumulative amount of compound permeated in the receiver compartment was plotted as a function of time. Experiments were performed in triplicate and results are presented as average ± S.D.

#### 2.2.8. Cell Culture

The human nasal cell line RPMI 2650 (CCL-30) was provided by Prof. Fabio Sonvico (University of Parma, Parma, Italy). Cells between passages 16–30 were grown at 37 °C in complete DMEM culture media supplemented with 10% (*v*/*v*) FBS and 1% (*v*/*v*) non-essential amino acid solution. Cells were grown in 75 cm^2^ culture flasks and maintained in a humidified atmosphere of 95% air and 5% CO_2_. Cells were sub-cultured according to the ATCC protocol. Cells were cultivated in 96-well tissue culture plates (5 × 10^3^ per well) and treated with different concentrations (0.012–5 mg/mL) of nanoemulsion for 72 h. Non-treated cells were used as control.

#### 2.2.9. In Vitro Cell Proliferation Assay

Inhibition of cell proliferation was assessed 72 h after treatment with nanoemulsions by the MTT assay according to the manufacturer’s standard protocol. MTT stock solution (5 mg/mL) was added to each culture being assayed to equal one tenth of the original culture volume and incubated for 3 h. At the end of the incubation, the solution was removed, and the insoluble formazan crystals were dissolved in 1:1 isopropanol/DMSO solution. The generated amount of blue formazan was measured at 570 nm by a spectrophotometer. Cell viability was calculated using the following equation:

(1) $$\mathrm{Cell}\;\mathrm{viability}(\%)=\frac{OD\;of\;treated\;cells}{OD\;of\;control}\times 100$$

where, *OD* is the optical density.

## 3. Results and Discussion

### 3.1. Oil-in-Water (o/w) Nanoemulsions

#### 3.1.1. Preparation of Nanoemulsions

As can be seen in Table 1, low-energy nanoemulsions (L1) were prepared with IPM as dispersed oil phase, water as continuous phase, a 2:2:1 blend of Tween 80/Labrasol/Maisine as surface-active agents and Transcutol as co-solvent. Then, low-energy nanoemulsions (L2) which contained a 1:4 mixture of IPM/EVOO as oil phase, water as continuous phase and the same blend of surfactants (Tween 80/Labrasol/Maisine) were formulated. Both low-energy nanoemulsions (L1, L2) were prepared with mild stirring at room temperature and remained optically clear for 60 and 22 days, respectively. Then, nanoemulsions having the same composition as L1 but homogenized by the two-step high-energy emulsification procedure were developed (H1). When IPM was replaced by EVOO and lecithin was added in place of Labrasol/Maisine in the surfactants’ mixture, mechanical agitation and high-pressure homogenization were applied to provide the necessary disruptive forces for the production of oil droplets at the nanoscale (H2). High-energy nanoemulsions (H1,H2) remained visually stable for 28 and 50 days, respectively.

In this study, all nanoemulsions contained Tween 80, a non-ionic surfactant widely used for the formulation of biocompatible o/w emulsions and nanoemulsions [36,37,38]. L1, L2 and H1 also contained a 2:1 mixture of the non-ionic surfactants Labrasol (caprylocaproyl polyoxyl-8 glycerides) and Maisine (glyceryl monolinoleate). Caprylocaproyl polyoxyl-8 glycerides have been reported as appropriate surfactant molecules for the formulation of biocompatible o/w nanoemulsions in several studies [39,40]. Maisine is a mixture of monoglycerides, mainly glyceryl monooleate and glyceryl monolinoleate, together with variable quantities of diglycerides and triglycerides, obtained by partial glycerolysis of vegetable oils.

It has been well established in the literature that mutual solubilization of two immiscible liquids such as water and oil increases when nonionic surfactants with different hydrophilic lipophilic balance (HLB) values are mixed [41,42]. In the present study, the HLB value of the mixture of Tween 80/Labrasol/Maisine was calculated by adding values of the individual surfactants and was found equal to 11. In an attempt to formulate stable o/w nanoemulsions, soybean lecithin, a biocompatible and biodegradable emulsifier widely used in foods, cosmetics and pharmaceuticals, was selected [5,39,43]. The HLB of the Tween80/Lecithin mixture was calculated as mentioned above and was found equal to 14. L1 and H1 also contained Transcutol (diethylene glycol monoethyl ether), a powerful solubilizer used as co-solvent in many surfactant formulations.

After preparation, the nanoemulsions were studied using analytical techniques based on scattering and viscometry in order to clarify their characteristics in relation to their composition and method of preparation.

#### 3.1.2. Droplet Size and PdI

Mean droplet diameter and PdI of the nanoemulsions were obtained using DLS, and the results are shown in Table 2. Both low-energy nanoemulsions (L1, L2) showed droplet sizes < 80 nm and PdI < 0.150. In the case of L1 system, the mean droplet diameter was twice the value of the droplet diameter of L2 system. This finding indicates that the presence of EVOO in the oil phase and the presence of Transcutol as co-solvent affect the size and the size distribution of the oil droplets when the nanoemulsions were stabilized with 5% *w*/*w* of the same surfactants’ mixture. When nanoemulsions emulsified with the high-energy technique (H1, H2) were tested, much larger oil droplets were observed. More specifically, nanoemulsions having the same composition as L1 ones and prepared by the high-energy technique (H1) showed hydrodynamic diameter of 142.9 ± 4.6 nm and low polydispersity (<0.1). Then, nanoemulsions formulated with Tween 80, Lecithin and EVOO (H2) showed the highest hydrodynamic diameter (220.5 ± 4.8 nm) and the highest PdI (0.230 ± 0.007) of all nanoemulsions examined in this study. Moreover, due to the nature of the surfactants used to formulate the o/w nanoemulsions, the surface of the dispersed oil droplets was not charged as evidenced by ζ-potential measurements. In all systems, values of ζ-potential around zero were obtained (data not shown).

#### 3.1.3. Stability

**Figure 1:**
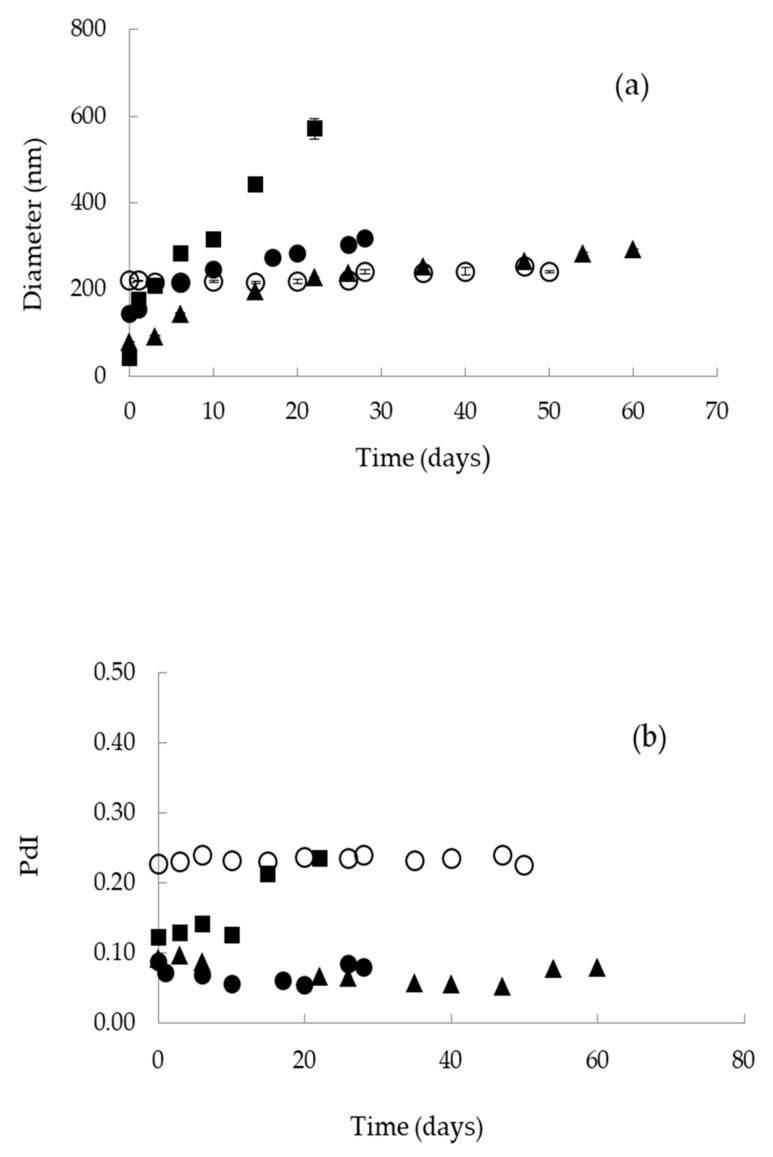
Storage stability of o/w nanoemulsions: (**a**) Droplet size and (**b**) polydispersity index (PdI) as a function of time. (▲): L1; (■): L2; (●): H1; (○): H2.

Following, the stability of the nanoemulsions was evaluated by comparing their mean droplet diameter and PdI over time at constant temperature (25 °C) using DLS (Figure 1a,b). As can be seen, droplet size of L1 nanoemulsions was increased from 79 to 200 nm within the first 15 days and then continued to increase slowly reaching 300 nm after a total of 60 days. L2 nanoemulsions were characterized by a sharp increase of droplet size from 42 to 571 nm within 22 days, followed by phase separation. H1 nanoemulsions were also destabilized after droplet size increase within 28 days. Finally, oil droplets of H2 nanoemulsions remained relatively large (>200 nm) but stable for 50 days before phase separation (Figure 1a). The uniformity of the nanoemulsions as expressed by PdI was not affected with storage, except system L2 which was rapidly destabilized 22 days after preparation due to droplet size heterogeneity (Figure 1b).

Although L1, L2 and H1 nanoemulsions were prepared keeping the same surfactant concentration (5% *w*/*w*) and the same o/w weight ratio (0.02), their stability with time was different (Figure 1a,b). This result could be associated with the absence of co-solvent and also the addition of EVOO in oil phase of the L2 nanoemulsions resulting in higher polydispersity. In some cases, the presence of co-solvents can induce increased stability as a result of adsorption and conformation changes at the o/w interface and also physicochemical changes of the bulk phase [44,45]. On the other hand, H2 nanoemulsions were prepared in the absence of co-solvent, at lower surfactant concentration, at higher o/w weight ratio (0.04), and also higher HLB value of the surfactants mixture. These nanoemulsions remained stable for 50 days, despite having the highest droplet size (220.5 ± 4.7 nm) and highest PdI (0.230 ± 0.007) of all systems examined.

#### 3.1.4. Viscosity

The viscosity of all prepared nanoemulsions (L1, L2, H1, H2) was measured and found in the range of 1.24–1.50 centipoise (cP) as can be seen in Table 2. Similar viscosities were obtained when o/w nanoemulsions consisting of 92% (*w*/*w*) aqueous phase, 3.6% *w*/*w* EVOO and surfactant mixtures were tested. Those o/w nanoemulsions were developed and characterized for the encapsulation of vitamin D_3_ [39]. In the present study, it was shown that the nature of the dispersed oil phase affects the viscosity of the nanoemulsions. When IPM was replaced by EVOO, an increase of the viscosity up to 1.50 ± 0.02 cP was observed. In general, the viscosity of emulsions is a function of the components (oil, water, surfactants) and their concentrations. Interestingly, nanoemulsions with the same composition but different droplet size (L1 and H1), due to the different emulsification procedure, had different viscosities. This is consistent with observations from the literature were droplet size of nanoemulsions was related to the viscosity of the continuous phase and also the viscosity of the dispersed oil phase [46].

Taking into consideration the findings mentioned above, L1 nanoemulsions consisted of 92% *w*/*w* water, 2% *w*/*w* IPM, 5% w/w blend of surfactants (Tween 80/Labrasol/Maisine, 2:2:1) and 1% *w*/*w* Transcutol, formulated with the low-energy homogenization technique were proved as more appropriate for the encapsulation of lipophilic compounds. L1 nanoemulsions had very low droplet size and PdI, their viscosity is the lowest of all systems examined and remained structurally stable for at least 60 days. What is more, L1 nanoemulsions contained Transcutol a co-solvent used as solubilizing agent for all types of dosage forms and also known as topical penetration and permeation enhancer.

### 3.2. Encapsulation of Lipophilic Compounds in o/w Nanoemulsions

Lipophilic compounds of pharmacological interest such as vitamin D_3_ and curcumin were encapsulated into the oil cores of low-energy o/w nanoemulsions (L1) as described in Methods (see Section 2.2.1). The effect of their presence on droplet size, polydispersity, stability and membrane dynamics is of great importance for their potential application as biocompatible nanocarriers. These properties were investigated by EPR spectroscopy and DLS.

#### 3.2.1. Droplet Size, PdI and Stability

- Vitamin D_3_

**Figure 2:**
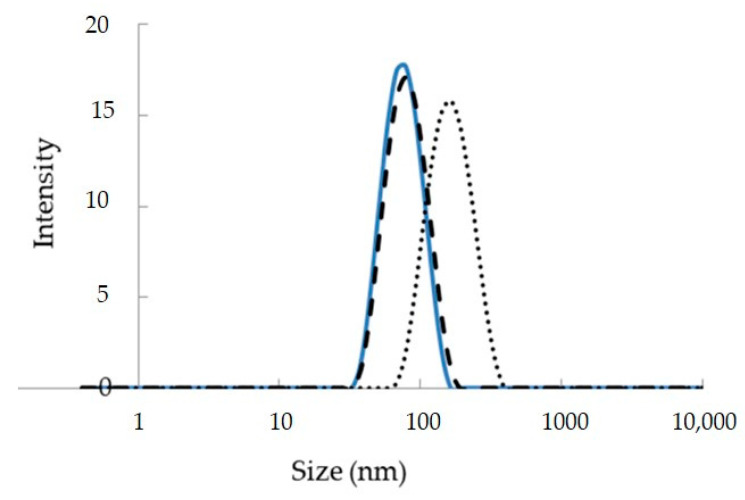
Droplet size distribution of empty and loaded L1 nanoemulsions. Encapsulation of vitamin D_3_ and curcumin in low-energy o/w nanoemulsions based on water, IPM, Tween 80, Labrasol, Maisine and Transcutol (L1). (─): empty, (- - -): loaded with vitamin D_3_, (…): loaded with curcumin.

**Figure 3:**
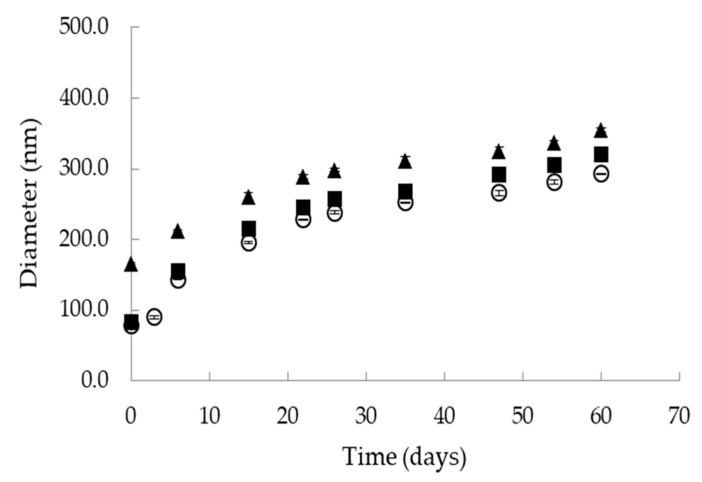
Storage stability of o/w nanoemulsions based on water, IPM, Tween 80, Labrasol, Maisine and Transcutol (L1). (○): empty, (■): loaded with vitamin D_3_, (▲): loaded with curcumin.

DLS measurements of L1 nanoemulsions showed that vitamin D_3_ addition in the dispersed oil phase caused a small increase in the size of the oil droplets from 78.6 ± 0.2 nm to 83.6 ± 0.3 nm (Figure 2). The PdI was slightly increased from 0.092 ± 0.003 to 0.102 ± 0.005. Similar behaviour has been observed when vitamin D_3_ was encapsulated in o/w nanoemulsions based on water, EVOO and a 10:1 mixture of Tween 20 with lecithin [39]. In a previous study of our group, vitamin D_3_ was added in the oil cores of 3 different o/w nanoemulsions formulated with water, edible oils and Tween 20 or Tween 20/lecithin mixtures as emulsifiers. In this study, the size of the oil droplets was increased upon vitamin D_3_ encapsulation in a concentration dependent way [5]. This behaviour could be possibly related to vitamin’s interaction with the surfactants layer affecting both interfacial tension and overall emulsion stabilization. Storage stability of vitamin D_3_ loaded nanoemulsions at 25 °C was tested by DLS analysis of droplet size over time (Figure 3). Although the diameter of the oil droplets was gradually increased with time up to 320 nm, the nanoemulsions remained physically stable for 60 days.

- Curcumin

DLS measurements of L1 nanoemulsions loaded with curcumin showed an increase of droplet size from 78.6 ± 0.2 nm to 165.6 ± 1.0 nm and also a decrease of PdI from to 0.092 ± 0.003 to 0.072 ± 0.008 (Figure 2). When storage stability of curcumin-loaded nanoemulsions was tested by DLS analysis of droplet size over time, stable nanoemulsions for 60 days were obtained (Figure 3). Α recent study of microstructural investigation of curcumin-loaded low-energy nanoemulsions stabilized by polysorbate 80 and soybean lecithin indicated interfacial location of the active compound [30]. In this study, droplet size growth with increase in the concentration of curcumin was also observed. In another study of the same group, low-energy nanoemulsions were characterized as carriers of curcumin with reference to dermal administration. Comparative physicochemical properties of the empty and drug-loaded nanoemulsions showed that curcumin presence in the formulation induced significant particle size augmentation [47].

#### 3.2.2. Dynamics of the Surfactant Layer

To obtain information on the dynamics of the surfactant layer, EPR spin probing was applied in empty and loaded nanoemulsions. For this purpose, 5- and 16-DSA were used as amphiphilic spin probes. These molecules are spin-labelled fatty acid analogues consisting of stearic acid and an N–O· moiety attached to the C-5 and C-16 position of the hydrocarbon chain, respectively. Due to their amphiphilic nature, both spin probes are located at the oil/water interface interacting with the surfactant molecules.

**Figure 4:**
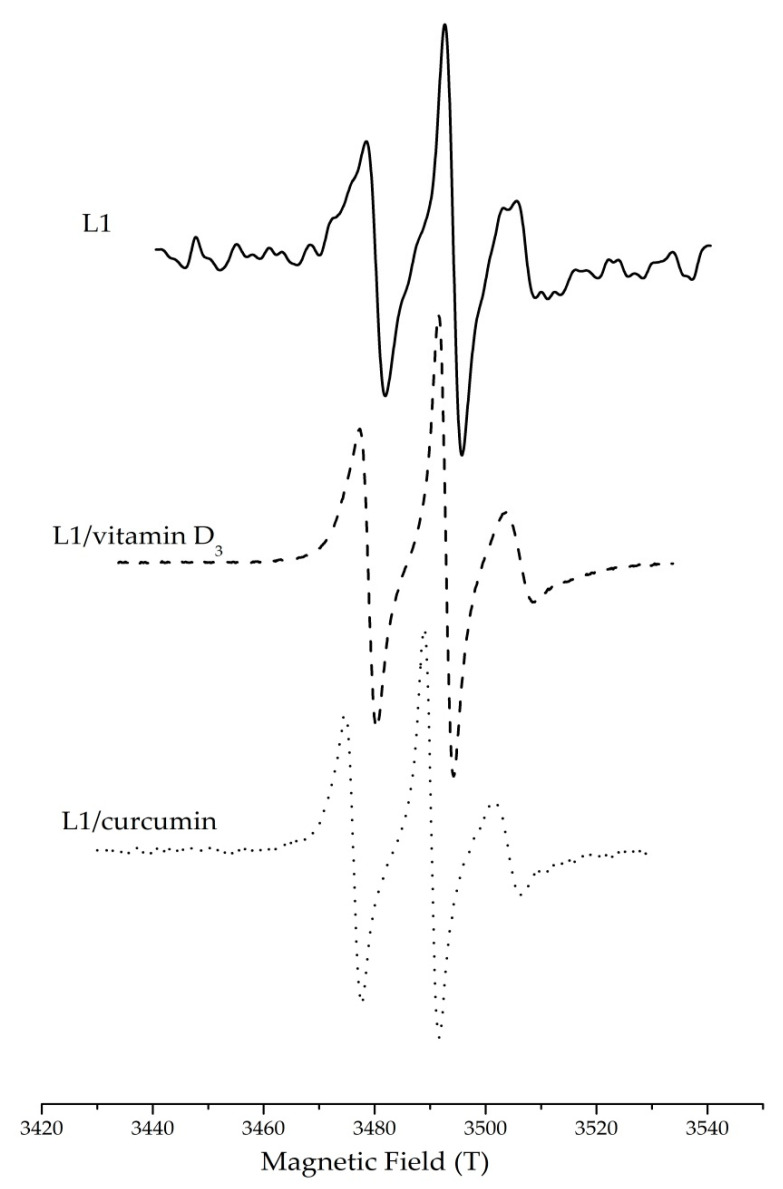
EPR spectra of 5-DSA in o/w nanoemulsions based on water, IPM, Tween 80, Labrasol, Maisine and Transcutol (L1) in the presence and absence of vitamin D_3_ and curcumin.

Mobility, order and polarity across the surfactant monolayer in the nanoemulsions were detected through analysis of the corresponding EPR spectra. Figure 4 demonstrates the characteristic three-line EPR spectra of doxyl derivatives (5-DSA) in empty and loaded o/w nanoemulsions. The observed EPR spectra are characterized by unequal line heights and line widths indicating restrictions in the mobility of the spin probe in the surfactant layer. Rotational correlation time (τ_R_), order parameter (S) and isotropic hyperfine coupling constant (α_N_) were calculated from the EPR spectra as reported elsewhere [33,34]. The results of the EPR study are shown in Table 3.

The paramagnetic moiety of 5-DSA is localized in the part of the surfactants monolayer that is closer to the polar head groups while 16-DSA is in a deeper part of the same monolayer closer to the dispersed oil phase. The higher τ_R_ and S values of 5-DSA in comparison to the corresponding ones of 16-DSA indicate that 5-DSA is located in a region where the surfactants are organized in a more tight way in contrast to the more flexible environment near the oil phase where 16-DSA is located.

The hyperfine coupling constant (α_Ν_) is directly related to the distance between peaks in the EPR spectra, and its magnitude indicates the extent of spin delocalization and spin polarization of the unpaired electron. In the present study, the hyperfine coupling constants (α_N_) of 5- and 16-DSA were calculated to investigate the polarity profile across the surfactant monolayer. As can be observed, in the case of 16-DSA, hyperfine coupling constants were higher as compared to these of 5-DSA in both empty and loaded nanoemulsions (Table 3). In fact, hyperfine coupling constants of 16-DSA are in all cases comparable to these of pure solvent, namely IPM, verifying the findings mentioned above concerning location of 16-DSA near the oil phase [48].

- Vitamin D_3_

In the case of 5-DSA, addition of vitamin D_3_ in the o/w nanoemulsions caused an increase of τ_R_ from 2.73 ns to 3.62 ns and a decrease of order parameter from 0.16 to 0.11. The latter could be related to the increase of droplet size upon vitamin addition and the consequent decrease in the curvature of the surfactants layer. When the curvature is reduced, the hydrocarbon chains of the surfactants are packed tighter, thus hindering the motion of the amphiphilic spin probes. In other words, addition of vitamin D_3_ in the o/w nanoemulsions increased the rigidity of the surfactants layer. The polarity as expressed by α_N_ remained the same, 13.8 mT, indicating that the nitroxide group of the spin probe is localized in the hydrophobic environment of the surfactant tails. Similar behaviour has been reported for the encapsulation of vitamin D_3_ in edible o/w nanoemulsions developed for various food applications [5,39]. In the case of 16-DSA, neither τ_R_ nor S nor α_N_ were affected by the presence of vitamin D_3_ which can be related to negligible interaction of the added compound with the spin probe at the depth of its location in the monolayer.

- Curcumin

When curcumin was encapsulated in the nanoemulsions, both τ_R_ and order parameter S were decreased as compared to the empty systems (Table 3). The observed decrease in the order parameter S from 0.16 to 0.11 is probably due to the increase of droplet size upon curcumin addition and the consequent decrease in the curvature of the surfactants monolayer. The decrease of τ_R_ for both spin probes indicates that curcumin affects the movement of the spin probes regardless the depth of their location in the membrane. It seems that curcumin molecules, probably due to their chemical structure (symmetric molecules having two aromatic ring systems and a bent conformation) are making free space among tightly packed surfactant molecules, thus enabling both 5-DSA and 16-DSA to rotate more freely [49]. This phenomenon is more pronounced in his case of 5-DSA, a spin probe with the N–O· moiety closer to the surfactants polar head groups. According to the results of the EPR study, curcumin appears to be closer to the polar region of the surfactant monolayer. Obviously, if curcumin was completely solubilized in the oil phase or located closer to the surfactants’ nonpolar tails, more pronounced changes in 16-DSA dynamics would be detected.

These findings are in accordance with previous microstructural investigation of low energy o/w nanoemulsions also indicating interfacial location of curcumin [31]. Local polarity was not affected by the presence of curcumin since the oil soluble molecules did not displace the spin probes in an environment with different polarity.

### 3.3. Nanoemulsion-Based Hydrogels

#### 3.3.1. Preparation of Nanoemulsion-Based Hydrogels

A series of hydrogels based on natural polymers were prepared as mentioned in Methods (see Section 2.2.2). Changes in physical characteristics (colour, texture and appearance) of the hydrogels during storage at ambient temperature were observed visually. The xanthan-gum-based hydrogels were separated shortly after preparation. The HPMC based hydrogels remained physically stable for 25 days and then were destabilized due to water separation from the polymer network. On the contrary, the chitosan-based hydrogels remained stable for a time period of at least three months. Therefore, considering their physical stability as well as the valuable properties of chitosan as biomaterial (biodegradability, lack of toxicity, anti-fungal effects, and immune system stimulation), hydrogels consisting of chitosan were selected for further studies.

For the development of nanoemulsion-based hydrogels, tests with various concentrations of chitosan and mixtures of acetic acid solution (1%)/nanoemulsion at different mixing ratios were performed. The nanoemulsions used for the development of these specific hydrogels were formulated with the low-energy homogenization procedure and consisted of 92% *w*/*w* water, 2% *w*/*w* IPM, 5% *w*/*w* blend of surfactants (Tween 80/Labrasol/Maisine, 2:2:1) and 1% *w*/*w* Transcutol (L1 system). Finally, a system containing 1.25% *w/v* chitosan in a 1:1 mixture of acetic acid solution (1%)/nanoemulsion, was selected as the most appropriate. The selection was based on physical stability of the hydrogels.

For the encapsulation of lipophilic compounds in hydrogels, nanoemulsions loaded with vitamin D_3_ (0.06 mg/g) or curcumin (0.06 mg/g) were initially formulated as described in Methods (see Section 2.2.1). These loaded nanoemulsions were then mixed with acetic acid 1% and chitosan as mentioned above, resulting in the nanoemulsions-based hydrogels. The concentration of the lipophilic compounds in the nanoemulsion-based hydrogels was 0.03 mg/g.

#### 3.3.2. Dynamics of the Surfactant Layer in Nanoemulsion-Based Hydrogels

Dynamics of surfactant monolayers in nanoemulsion-based hydrogels were investigated by EPR spin probing spectroscopy, and the results are presented in Table 3. As mentioned above (see Section 3.2.2), the presence of vitamin D_3_ and curcumin affected both rotational correlation time (τ_R_) and order parameter of the loaded nanocarriers, indicating interfacial location. Interestingly, membrane dynamics in nanoemulsion-based hydrogels as expressed by τ_R_, S and α_N_ were affected in a similar way as in nanoemulsions. This finding indicates that chitosan used as gelling agent for nanoemulsion thickening at a concentration of 1.25% *w/v* affects neither the rigidity nor the polarity of the surfactants’ monolayer. In other words, during the sol-to-gel transition of the nanoemulsions at ambient temperature, chitosan neither adsorbs onto the droplet interface nor interacts with the nonionic surfactants as it has been reported for other gelling agents such as small molecules or triblock copolymers [50].

To summarize, low viscous o/w nanoemulsions were formulated with the low-energy emulsification technique, using water, IPM as the dispersed oil phase, Transcutol as co-solvent and a mixture of Tween 80/Labrasol,/Maisine as surfactants. Then, lipophilic bioactive compounds such as vitamin D_3_ and curcumin were added in the oil phase and dispersed in water with the help of the surfactants. Encapsulation of the bioactive compounds caused an increase of droplet size; nevertheless, both empty and loaded nanoemulsions remained stable for two months as evidenced by DLS measurements of droplet size and polydispersity index. Interfacial properties of the nanoemulsions were investigated in terms of spin probes mobility and order using EPR spin spectroscopy and amphiphilic lipid spin probes. EPR results clearly showed that solubilization of vitamin D_3_ and curcumin influenced the rigidity and order of the membrane indicating interfacial location of the compounds. Then, the nanoemulsions were gellified with the addition of chitosan to result nanoemulsion-based hydrogels. Rigidity and order of the surfactant monolayer in the hydrogels were tested with EPR spin probing spectroscopy to investigate the effect of the gellifying procedure. Interestingly, addition of chitosan as a gelling agent did not affect membrane dynamics.

Then, to evaluate the ability of the proposed nanoemulsions and nanoemulsion-based hydrogels to serve as effective carriers of vitamin D_3_ and curcumin, in vitro release studies were performed.

### 3.4. In Vitro Release Study

In vitro release experiments were started by placing a certain amount of each loaded nanocarrier in the donor compartment of the cell. The in vitro release profiles of vitamin D_3_ and curcumin from the formulations were investigated for 26 and 28 h, respectively, in 1:1 PBS buffer/ethanol solution (1:1).

**Figure 5:**
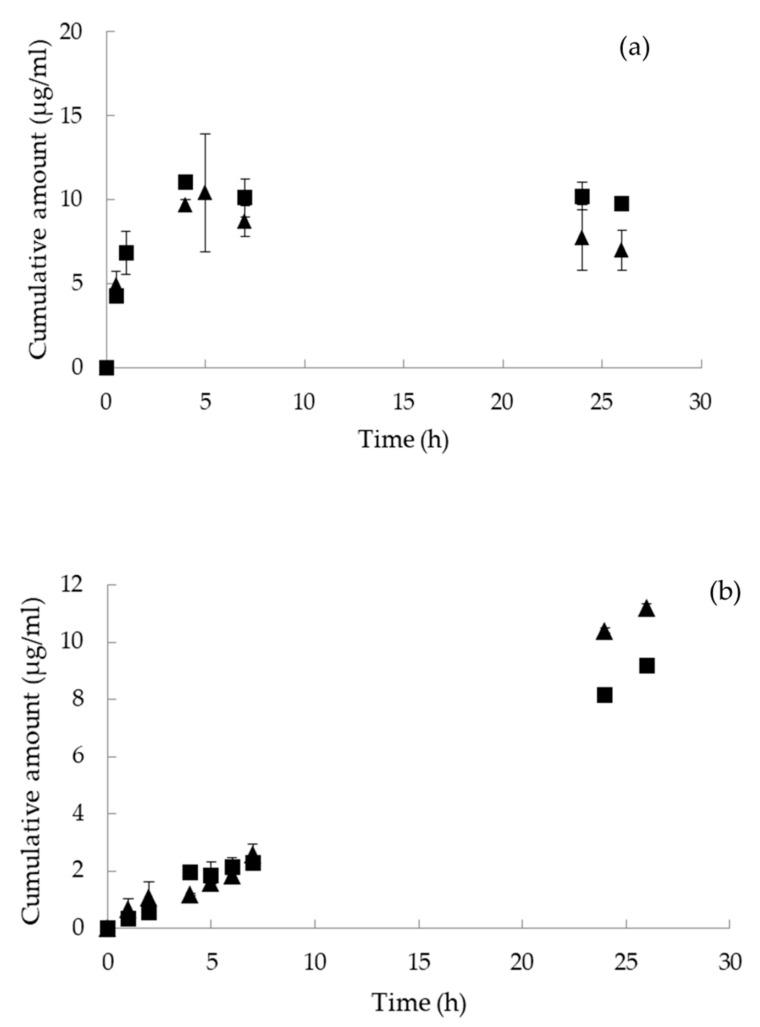
In vitro release of encapsulated (**a**) vitamin D_3_ and (**b**) curcumin from (▲) nanoemulsions and (■) nanoemulsion-based hydrogels at 32 ± 0.5 °C.

In all cases, an increase of vitamin D_3_ and curcumin in the receptor chambers with time was observed. More specifically, 8% of curcumin was released from the nanoemulsions and 13% from the nanoemulsion-based hydrogels in 28 h. Curcumin release occurred, from both systems, according to the zero-order kinetic described with a linear relationship between the cumulative amount of the released drug with time. In the case of vitamin D_3_, up to 12% was released from the nanoemulsions and 33% from the nanoemulsion-based hydrogels in 26 h. The release of vitamin D_3_ is divided into 2 stages according to the release rate as indicated by the slope of the release profile. Initially, a burst release was observed for the first 5 h (Figure 5). Then, the release rate was significantly decreased for the second stage, i.e., after day 5 and during the next 11 days. As can be observed from the plots of Figure 5, the release rate of both lipophilic compounds from the nanoemulsions was comparable to the corresponding ones from the nanoemulsion-based hydrogels at 32 ± 0.5 °C. According to the literature, release rate constants are depended on both the composition of the nanocarrier and the incorporated active compound [51]. In the case of micro- and nanoemulsions, the composition of the oil phase as well as the type of the emulsifiers affect the release of the encapsulated compounds. Hydrogels on the other hand possess a large amount of water rendering the encapsulation and release of hydrophobic compounds problematic. For this reason, the development of hydrogels that contain hydrophobic components such as hydrophobic polymers can improve the performance of these carriers [52]. In the present study, o/w nanoemulsions were added in the aqueous phase of the hydrogels to enable solubilization and release of vitamin D_3_ and curcumin. The observed differences in the release profiles were probably related to the nature and solubility of the encapsulated compound and not to the composition and physicochemical characteristics of the nanocarriers.

### 3.5. In Vitro Cell Viability

**Figure 6:**
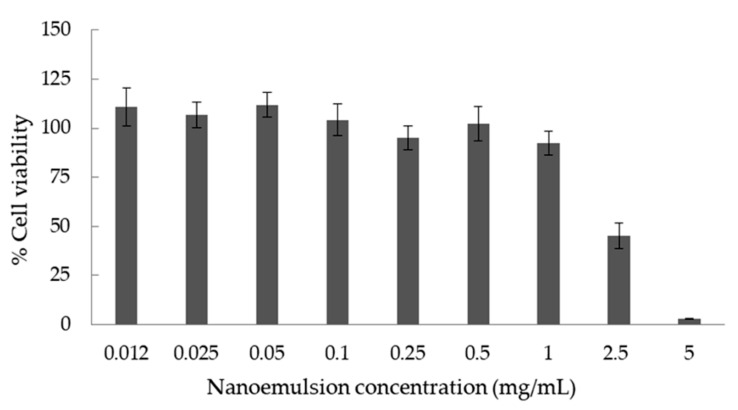
Cell viability testing results in RPMI 2650 nasal epithelial cell line after 72 h of treatment with L1 nanoemulsions using the MTT cell proliferation assay. Each column represents the mean of six replicates ± SD.

To assess the effect of L1 nanoemulsions on cell viability in vitro, the colorimetric MTT assay was performed. MTT is a tetrazolium-based assay which measures the reduction of tetrazolium salt by intracellular dehydrogenases of viable living cells. To perform this assay, RPMI 2650 nasal epithelial cells were treated with the nanoemulsions at a concentration range of 0.012 to 5 mg/mL in the culture medium. As can be seen from Figure 6, the nanoemulsions did not exhibit cytotoxic effect up to the concentration threshold of 1 mg/mL in the cell culture medium. However, further increase of the nanoemulsion concentration increased the cytotoxicity, a result that could be attributed to the increased concentration of the systems’ ingredients in the culture medium. It is known that surfactants, especially Tween 80, can disrupt the cell membrane of epithelial cells. As has been previously mentioned, Tween 80 significantly reduces the viability of RPMI 2650 cells in a dose-dependent way [53,54]. A similar cytotoxicity study was reported recently regarding the effect of a drug loaded o/w nanoemulsion on nasal epithelial cells RPMI 2650. More specifically, Espinoza et al. proposed o/w nanoemulsions based on a mixture of Labrasol/Tween 80/Transcutol P as surfactants and Capryol 90 as the oil phase for nose-to-brain delivery of Donepezil [55]. In that study, cell viability greater than 80% was observed when drug-loaded nanoemulsions were added in the cell culture at concentrations ranging from 3.125 to 25 µg/mL. It has to be underlined that the concentration of 1 mg/mL (Figure 6) is relatively high, in terms of in vitro assays, making the proposed system a promising carrier for the delivery of vitamin D_3_ and curcumin.

To conclude, the present research suggests that biocompatible o/w nanoemulsions and nanoemulsion-based hydrogels developed and structurally characterized as carriers of lipophilic compounds were suitable as vitamin D_3_ and curcumin delivery systems. These nanoemulsions and the nanoemulsion-based gels may hold promise as effective alternative for the intranasal delivery of various lipophilic bioactive molecules.

## Figures and Tables

**Table 1:** Composition of the nanoemulsions.

Ingredients (% *w*/*w*)	System			
	L1	L2	H1	H2
Water	92	93	92	92
Tween 80	2	2	2	4
Labrasol	2	2	2	-
Maisine	1	1	1	-
Transcutol	1	-	1	-
Lecithin	-	-	-	0.4
IPM	2	0.4	2	-
EVOO	-	1.6	-	3.6

**Table 2:** Mean droplet diameter, PdI and viscosity of the o/w nanoemulsions measured immediately after preparation.

System	Mean Droplet Diameter (nm)	PdI	Viscosity (cP)
L1	78.6 ± 0.2	0.092 ± 0.003	1.24 ± 0.01
L2	41.8 ± 0.3	0.122 ± 0.007	1.31 ± 0.04
H1	142.9 ± 4.6	0.090 ± 0.013	1.33 ± 0.02
H2	220.5 ± 4.7	0.230 ± 0.007	1.50 ± 0.02

**Table 3:** Rotational correlation time (τ_R_), order parameter (S) and isotropic hyperfine coupling constant (α_N_) of 5-DSA and 16-DSA in empty and loaded nanoemulsions (L1, L1/VD_3_, L1/CU) and nanoemulsions-based hydrogels (L1HG, L1HG/VD_3_, L1HG/CU).

System	5-DSA			16-DSA		
	τ_R_ (ns)	S	α_N_ (×10^−4^ T)	τ_R_ (ns)	S	α_N_ (×10^−4^ T)
L1	2.73	0.16	13.8	0.36	0.02	14.8
L1/Vitamin D_3_	3.62	0.11	13.8	0.35	0.04	14.7
L1/Curcumin	2.14	0.11	14.0	0.33	0.04	14.8
L1HG	2.73	0.17	13.6	0.37	0.04	14.6
L1HG/VD3	3.61	0.13	13.8	0.35	0.04	14.7
L1HG/Curcumin	2.23	0.11	14.0	0.36	0.04	14.7
